# Lower allergen levels in hypoallergenic Curly Horses? A comparison among breeds by measurements of horse allergens in hair and air samples

**DOI:** 10.1371/journal.pone.0207871

**Published:** 2018-12-12

**Authors:** Eva Zahradnik, Bente Janssen-Weets, Ingrid Sander, Benjamin Kendzia, Wolfgang Mitlehner, Caroline May, Monika Raulf

**Affiliations:** 1 Institute for Prevention and Occupational Medicine of the German Social Accident Insurance, Institute of the Ruhr-Universität Bochum (IPA), Bochum, Germany; 2 Luxemburg Institute of Health, Esch-sur-Alzette, Luxemburg; 3 Private Medical Practice Pneumology, Internal Medicine, Allergology, Klappholz, Germany; 4 Medizinisches Proteom-Center (MPC), Ruhr-Universität Bochum, Bochum, Germany; University of Illinois, UNITED STATES

## Abstract

**Background:**

Exposure to horses can cause severe allergic reactions in sensitized individuals. The breed, American Bashkir Curly Horse is categorized as hypoallergenic, primarily due to reports of allergic patients experiencing fewer symptoms while handling this special breed. The possible reasons for this phenomenon could be lower allergen production and/or reduced allergen release into the air because of increased sebum content in their skin and hair compared to other breeds. Therefore, the aim of the current study was to compare different horse breeds in relation to allergen content in hair and airborne dust samples.

**Methods:**

In total, 224 hair samples from 32 different horse breeds were investigated. Personal nasal filters were used to collect airborne dust during the grooming of 20 Curly Horses and 20 Quarter Horses. Quantitative analysis of all samples was performed using two newly developed immunoassays for the detection of horse dander (HD) antigens and the major allergen Equ c 1 and the commercial assay for Equ c 4. Results were analyzed using multiple linear regression models for hair samples and the Mann Whitney U test for airborne samples.

**Results:**

Horse antigen and allergen levels differed up to four orders of magnitude between individual animals. Despite enormous variability, levels of HD antigen, Equ c 1 and Equ c 4 in hair were significantly related to the breed and gender combined with the castration status of male animals. Curly Horses had significantly higher concentrations of all three tested parameters compared to the majority of the investigated breeds (medians: 11800 μg/g for HD antigen, 2400 μg/g for Equ c 1, and 258 kU/g for Equ c 4). Tinker Horses, Icelandic Horses and Shetland Ponies were associated with approximately 7-fold reduced levels of HD antigen and Equ c 1, and up to 25-fold reduced levels of Equ c 4 compared to Curly Horses. Compared to mares, stallions displayed increased concentrations of HD antigens, Equ c 1 and Equ c 4 by a factor 2.2, 3.5 and 6.7, respectively. No difference was observed between mares and geldings. No differences in airborne allergen concentrations collected with personal nasal filters during grooming were found between Curly and Quarter Horses.

**Conclusion:**

Breed and castration status had a significant influence on the antigen and allergen levels of horse hair. However, these differences were smaller than the wide variability observed among individual horses. Compared to other breeds, Curly Horses were not associated with lower allergen levels in hair and in air samples collected during grooming. Our approach provides no molecular explanation why Curly Horses are considered to be hypoallergenic.

## Introduction

Exposure to horses is considered a risk factor for the development of allergic rhinitis and asthma in susceptible (i.e. atopic) individuals [1,2]. Population-based studies conducted in Germany, Finland and Sweden indicated that the prevalence of horse sensitization among adults was 3.5%, 5.4% and 7.1%, respectively [3–5]. Allergy to horses is now also recognized as an increasing problem. The Swedish birth cohort study BAMSE recently reported an increase in sensitization to horses from 3% to 10% in 1699 children who were followed from 4 to 16 years [6]. The majority of horses in Europe today are used for leisure riding and competitive sports. Apart from persons who regularly handle horses, horse allergy can also affect people indirectly exposed to horse allergens because of their ubiquity in the environment [7]. In an Italian multicenter study, more than 50% of horse-sensitized patients denied any direct or indirect exposure to horses or horse owners [8]. In general, allergens from furred mammals easily become airborne, stick to human clothes and are transported to animal-free public spaces. For instance, horse allergens have been detected in schools [9], day care centers [10] and airplanes [11].

Four respiratory horse allergens are currently registered in the official list of allergens (http://www.allergen.org). The glycoprotein Equ c 1 is generally accepted as the major allergen and a highly predictive marker of allergy to horses. Component-resolved diagnostics studies have shown that up to 76% of patients with horse allergy react to Equ c 1 [12–14]. The sequence and crystal structure analysis of the recombinant form revealed that Equ c 1 is a dimeric molecule belonging to the lipocalin family [15,16]. Lipocalins represent the most important group of inhalant animal allergens and are primarily considered as pheromone and odorant carriers amongst a wide range of other biological functions. Equ c 2 has been also reported to be a lipocalin with a sensitization prevalence of 50%, but this allergen has not been cloned so far and only its N-terminal amino acid sequence is identified [17]. The serum albumin of horse, Equ c 3, is a minor allergen with sensitization rates of 18–30% [18,19]. The latherin Equ c 4 is a major component of horse sweat. It has strong surfactant properties and thermoregulatory function by moistening the waterproof pelt. It is the most likely cause of the frothing seen on sweating horses. The IgE binding frequency to Equ c 4 in 77% of horse sensitized subjects has been only reported in one study [20] and needs to be further evaluated. Equ c 4 is the only horse allergen that can be quantified by a commercial immunoassay (Equ c 4 ELISA, Indoor Biotechnologies). No detection systems are available for the other horse allergens.

**Fig 1:**
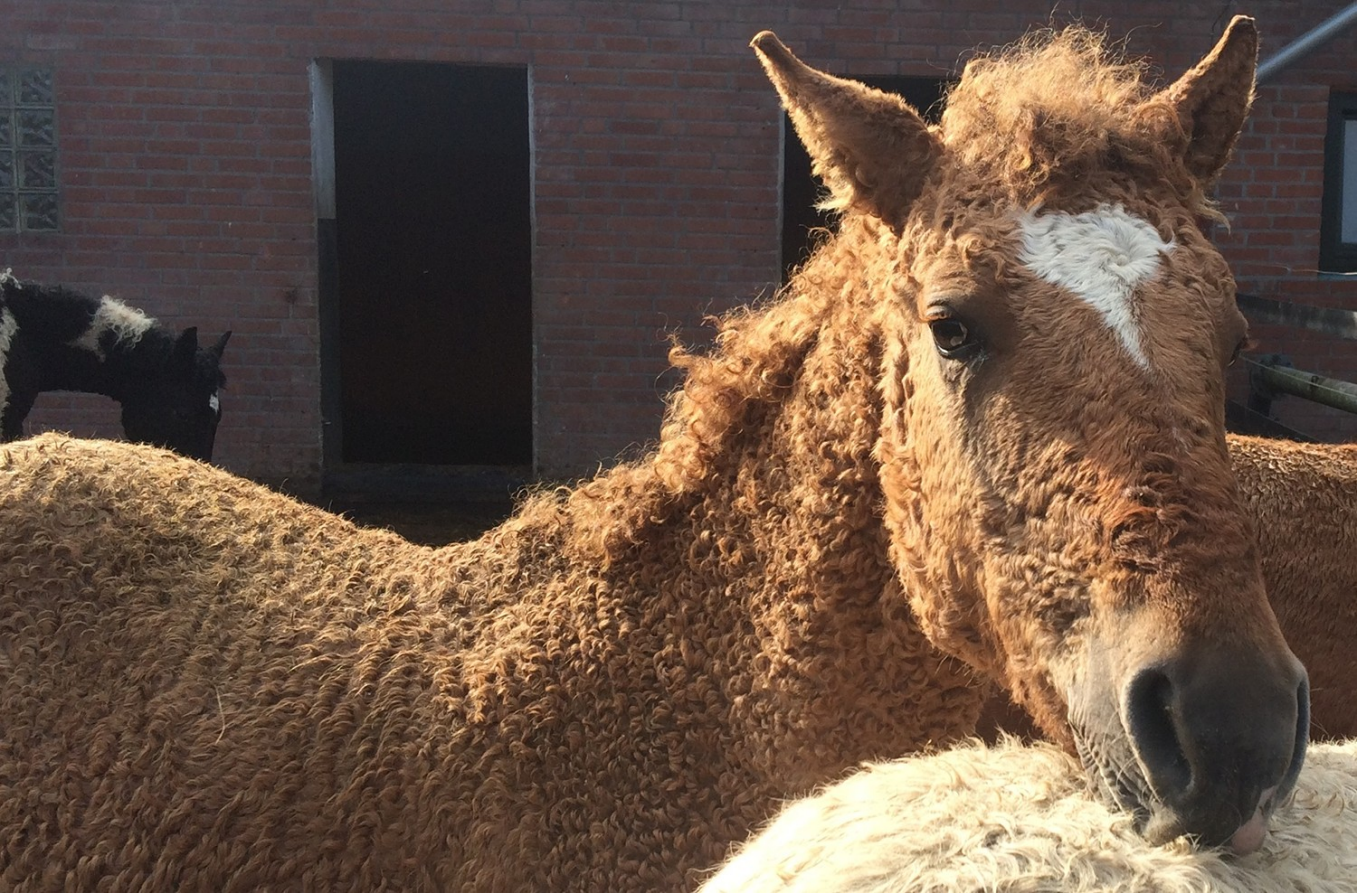
The American Bashkir Curly Horse.

There are more than 300 horse breeds in the world today. The American Bashkir Curly Horse (for short Curly Horse) is a very rare breed known for its unique curly coat of hair, curly mane and tail (Fig 1). This breed has been claimed as the only available hypoallergenic horse breed. This hypothesis is mostly based on experiences of persons allergic against horses who report no or less symptoms when riding Curly Horses. Some recent studies have shown that contact with these horses is possible without significant allergic reactions (examined by peak expiratory flow and lung function measurements in horse allergic patients) [21,22]. Moreover, regular riding and brushing of Curly Horses can induce clinical tolerance to other horse breeds. This supposed low allergenic potential of Curly Horses may be due to reduced content of allergens in horse epithelia [23]. In 1998, Felix et al. compared IgE-binding profiles of 20 pooled horse allergic patients’ sera to extracts of horse dander of several breeds, but did not find any significant differences in allergen composition between Curly Horses and other breeds [23]. Dander from all breeds investigated contained the most important allergens. The authors suggested that the reduced release of dander rather than its allergenic content may be the reason for the perceived low "allergenicity". Compared to other breeds, a very high sebum content was observed in the skin of Curly Horses. This could actually result in less spreading of allergens to the surroundings and reduced allergen quantities that are inhaled by allergic subjects. However, in the study of Felix et al. [23] only two extracts from Curly Horses were investigated and no quantitative measurements were performed.

The aim of this study was therefore to compare “hypoallergenic” Curly Horses to other horse breeds in relation to the allergen levels in hair samples. Allergen concentrations were determined using the commercial Equ c 4 ELISA and two newly developed immunoassays for the detection of horse dander (HD) antigens and the major allergen Equ c 1. In addition, allergen concentrations were measured in air samples that were collected using personal nasal filters to investigate the allergen release into the air during the grooming of Curly Horses and Quarter Horses.

## Material and methods

### Horse hair sampling

The collection of hair samples from different breeds of horses from 25 stables across Germany took place in March and April 2016 when horses normally change their coat. Hair was collected by grooming a horse with a new currycomb made of soft plastic. With the exception of the mane and the tail, hair was sampled from the entire horse’s body, mostly from the dorsum, flanks and neck. For each horse, breed, sex, castration status, age and main type of horse keeping/housing (pasture, tie-stall, box stall, paddock) was noted by the horse’s owner. In total, 224 hair samples were collected from 32 different horse breeds, including 106 mares, 92 geldings and 26 stallions (Table 1). To check the reproducibility of the results, 57 of the individuals whose hair was sampled in 2016 were re-sampled in 2017 during the same season.

**Table 1:** Population characteristics of the horses sampled in 2016.

Breed	Number [n]	Mares [n]	Geldings [n]	Stallions [n]	Age (years) [median (range)]
American Bashkir Curly Horse	33	23	4	6	8 (1–17)
Shetland-Pony	31	15	12	4	11 (1–26)
American Quarter Horse	23	16	4	3	6 (2–28)
German Riding Pony	22	9	12	1	18 (1–31)
Welsh Pony	18	8	3	7	9 (2–22)
Haflinger	13	4	9		18 (8–26)
Icelandic Horse	11	6	5		16 (4–24)
Oldenburg Horse	10	2	4	4	11 (4–20)
Westphalian Horse	9	3	6		9 (7–26)
Tinker Horse (Gypsy Vanner)	8	5	3		9 (5–25)
Hanoverian Horse	8	1	6	1	13 (6–19)
Trakehner	4	1	3		15 (10–15)
Norwegian Fjord Horse	4	2	2		24 (9–25)
Wielkopolski	3	2	1		14 (8–15)
English Thoroughbred	3	1	2		24 (18–25)
Andalusian Horse	3		3		12 (9–20)
Rhenish Warmblood	2	2			22 (18–25)
Dutch Warmblood	2		2		20 (7–32)
Holsteiner Horse	2	1	1		19 (14–23)
Friesian Horse	2		2		19 (13–24)
Connemara Pony	2	1	1		9 (8–9)
British Riding Pony	1	1			6
Fell Pony	1		1		22
Lewitzer Pony	1	1			5
Konik	1		1		5
Finnhorse	1	1			25
Arabian	1		1		16
Anglo-Arabian	1	1			23
Hispano-Àrabe	1		1		22
Arabian-Berber	1		1		21
Sachsen Anhaltiner	1		1		24
German Riding Horse	1		1		22
**Total**	**224**	**106**	**92**	**26**	**12 (1–32)**

According to the German Animal Welfare Act, the hair sampling was not an animal experiment and therefore no ethical approval was required. Hair was sampled during the grooming of horses, which is part of the regular horse care. Insofar no intervention or treatment of the horses associated with pain, suffering or damage was necessary to get the hair samples.

### Horse hair extraction

The extraction was performed by rotating 1 g of hair in 10 ml of phosphate-buffered saline (PBS; pH 7.4) plus 0.05% Tween 20 (PBST) for 1 h at room temperature. The extract was aspirated from the hair with a Pasteur pipette, transferred to a centrifugation tube and centrifuged for 20 min at 4°C and 20,000 x g. The supernatant was stored in aliquots at –80°C until analysis. The protein concentration was determined using the Bradford assay (Bio-Rad Protein Assay; Munich, Germany) with bovine serum albumin (BSA) as a standard.

### Horse dander extraction

Commercial horse dander substance was obtained from the manufacturer Allergon (Ångelholm, Sweden). The raw material was homogenized in PBST at 3000 rpm for 10 min (Schuett homogen-plus homogenizer, Göttingen, Germany) and centrifuged at 25000 x g for 15 min. The supernatant was stored in aliquots at –80°C until analysis. The protein content was determined by the Bradford assay. The horse dander extract was used as the antigen for the immunization and as the sandwich ELISA calibration standard.

### Production, purification and biotinylation of polyclonal antibodies

Polyclonal antibodies (pAbs) were raised in female New Zealand White rabbits immunized either with an extract of horse dander (Allergon) or with recombinant 6xHis-tagged Equ c 1 (expressed in yeast, affinity purified using IMAC) purchased from Flarebio, College Park, Maryland, USA (Cat. No. CSB-YP839158HO). Immunization was conducted by Charles River (Kisslegg, Germany) using four subcutaneous antigen injections of 0.2 mg each, according to the standardized protocol of the company. The final bleeding and serum collection was carried out 70 days after the first antigen injection.

For the antibody purification, the IgG fractions of both sera were isolated by affinity chromatography with HiTrap protein G columns (GE Healthcare, Uppsala, Sweden) following the manufacturer’s instructions. One part of each of the purified pAbs was used as capture antibodies in sandwich ELISAs. Another part was labeled with biotin and used as detection antibodies. Biotinylation was carried out by incubating the antibodies with a 33-fold molar excess of EZ-Link NHS-LC-Biotin (Thermo Scientific, Rockford, IL, USA) under continuous agitation for 2 h at room temperature. The biotinylated antibodies were dialyzed extensively with PBS to remove free biotin molecules.

### Purification of natural Equ c 1

Natural Equ c 1 was isolated from in-house horse hair extract (mixture of extracts from 193 individual horses of different breeds) by affinity chromatography with a HiTrap NHS column (GE Healthcare) coupled with 3 mg of the protein G-purified rEqu c 1-pAbs. The ligand coupling and purification procedure were carried out following the manufacturer’s instructions (GE Healthcare). The purity of the allergen preparation was verified by sodium dodecyl sulfate polyacrylamide gel electrophoresis (SDS-PAGE) and silver staining as previously described [24]. The eluted allergen was concentrated using Centriprep YM-10 (Millipore Corporation, County Cork, Ireland). The protein concentration was determined by the Bradford assay and by amino acid analysis. Both methods yielded same values of 0.14 mg/ml. The natural Equ c 1 was used as a calibration standard in the Equ c 1 sandwich ELISA.

### Amino acid analysis

The protein concentration determination was performed in duplicates by amino acid analysis as described by Plum et al. [25] with slight modifications. In short, 8 μl of the sample were dried in glass vials using a vacuum centrifuge (RVC 2–25 CD plus; Martin Christ Gefriertrocknungsanlagen GmbH, Germany). Afterwards, 6 M hydrochloric acid as well as one phenol crystal was added and the samples hydrolyzed by incubating for 1 h at 150°C in airtight tubes under argon atmosphere. Then 10 μL of 20 mM hydrochloric acid were added to each vial and 10 μL of this solution was further diluted with 30 μL borate buffer (inclusive 10 pmol N-valine for internal calibration) and 10 μl AccQ-Tag derivatization reagent. After an incubation at 56°C for 10 min, separation and quantification of the modified amino acids was performed with an ACQUITY UPLC system (Waters GmbH, Germany).

### Horse dander sandwich ELISA

MaxiSorp microtiter plates (Thermo Scientific, Roskilde, Denmark) were coated overnight at 4°C with protein G-purified horse dander pAbs in 0.1 M carbonate-bicarbonate buffer (pH 9.6) at 1.2 μg/mL (100 μL/well). After blocking with 1.5% casein in PBST (200 μL/well), the plates were incubated with standards, assay controls and samples diluted in PBST (100 μL/well). A horse dander extract (Allergon) was used as the standard, with concentrations ranging from 0.04 to 10 ng/mL. An extract of settled dust from a horse stable was used as positive control, and a wheat flour extract was used as a negative control. Each hair sample was tested using three serial dilutions. Biotinylated pAbs were added at 0.3 μg/mL PBST (100 μL/well), followed by incubation with peroxidase-streptavidin-conjugate (Poly-HRP80-SA, Fitzgerald, Acton, MA, USA) diluted 1:10000 in PBST. All incubations were carried out for 1 h at 22°C and followed by three washes with PBST between successive steps. Finally, the plates were developed with ABTS tablets [2,2’-azino-bis(3-ethylbenzothiazoline-6-sulfonic acid) diammonium salt; Sigma-Aldrich, Steinheim, Germany] diluted in 50 mM phosphate-citrate buffer (pH 4.2) with 0.015% hydrogen peroxide (100 μL/well). The reaction was stopped with 0.32% sodium fluoride (100 μL/well), and the optical density (OD) was read at 414 nm. The standard curves were obtained by 4-parameter curve fitting using SoftMax Pro 5.4.5 (Molecular Devices, Sunnyvale, CA, USA). The lower limit of detection (LOD) was defined by adding 0.1 OD units to the minimal value of the four-parameter curve fit function (parameter A). The upper limit of detection was the concentration corresponding to OD_414_ = 3.0. Allergen concentrations were calculated as the mean of the three dilution-adjusted measurements per sample. Results are given in micrograms per gram of hair.

### Equ c 1 sandwich ELISA

The Equ c 1 ELISA was developed following the design of the horse dander ELISA with the following modifications. The capture Equ c 1-pAbs were coated at 0.33 μg/mL and the biotinylated Equ c 1-pAbs were used at 0.16 μg/mL. The standard curve was obtained using the affinity-purified natural Equ c 1, with concentrations ranging from 0.02–5 ng/mL.

### Equ c 4 sandwich ELISA

Equ c 4 concentrations were quantified using an ELISA kit based on monoclonal antibodies (mAb) and horse hair extract as the calibration standard (Indoor Biotechnologies Inc., Charlottesville, VA, USA). The original ELISA protocol was modified with the aim to obtain higher sensitivity. The capture mAb 103 and the biotinylated detection mAb 14G4 were used at 1:1250 dilutions. The plates were blocked with 1% BSA in PBST. The standard was used in a concentration range of 0.002–0.5 U/mL. The recommended streptavidin-peroxidase-conjugate (Sigma S5512) was exchanged by 1:10000 diluted Poly-HRP80-SA (Fitzgerald, Acton, USA). All other reagents, buffers, and incubation steps were used and performed according to the protocol of horse dander sandwich ELISA as described above. The LOD was defined as parameter A + 0.05 OD units. Results are given in kilo units per gram of hair.

### Specificity analysis

The assay specificity was examined using extracts from mammalian epithelia (cat, dog, cow, rabbit, mouse, rat, guinea pig, hamster, goat, sheep, swine), mites (*Acarus siro*, *Lepidoglyphus destructor*, *Tyrophagus putrescentiae*, *Glycyphagus domesticus*, *Blomia tropicalis*, *Dermatophagoides farinae*, *D*. *pteronyssinus* and *D*. *microceras*), molds (*Alternaria alternata*, *Aspergillus fumigatus*, *A*. *niger*, *Cladosporium herbarum*, *Penicillium chrysogenum*, *Phoma betae* and *Bortrytis cinerea*) and grains (wheat, rye, barley, oat, corn and soy). The extracts were prepared according to the extraction procedure used for horse dander. The mammalian epithelia, mite and mold raw materials were obtained from Allergon or Greer (Lenoir, NC, USA), and the cereal grains were bought from a health food shop. For extraction, grains were first crushed in a mortar with the addition of liquid nitrogen.

### Nasal filter sampling

Personal nasal filters (Rhinix, Aarhus, Denmark) were used to collect inhaled airborne dust during grooming of the horses. Nasal filters were originally developed to prevent hay fever symptoms. The butterfly-shaped devices consist of low–air resistance interception membranes that are mounted on a copolymer frame. They are inserted directly into the nostrils and remove dust particles from the breathing air.

The sampling took place at the Riverside Curly Horse Ranch in Kamp-Linfort, North Rhine Westphalia, Germany. This ranch is the largest breeding farm of purebred Curly Horses in Europe. All horses are listed in the ABCR register (American Bashkir Curly Horse Registry). Besides Curly Horses, American Quarter Horses are also housed at the ranch. Both breeds are mainly trained for a special style of horseback riding named "Western Riding".

The nasal filters (size L) were worn by two subjects for 10 minutes during grooming. In total, 40 samples were collected during grooming. Each subject handled 10 Curly Horses and 10 Quarter Horses. To avoid a possible gender effect, both breed groups consisted of 13 mares, 4 stallions and 3 geldings. To avoid the detection of airborne horse allergens derived from other horses inside the stall buildings, the sampling took place in two separate rooms that were cleaned beforehand (one for each breed). In addition, 14 nasal filter samples were collected without grooming at different locations both inside and outside of the horse farm to determine the ambient allergen concentrations. Before insertion of the nasal filters, the subjects were instructed to blow their noses. During insertion and removal, nasal filters were handled with gloves on the crossbar at the middle to avoid contamination of the membranes. A mirror was used to check that the device was positioned correctly in the nose.

The two filter membranes were removed with tweezers from the plastic rack of the nasal filter and transferred to a 2 ml centrifuge tube with 1 ml PBST. The samples were incubated for 2 h at room temperature on a roller mixer. The membranes were then removed from the solution with clean tweezers and squeezed out at the top of the tube. The extracts were centrifuged for 10 min at 30000 x g and 8°C. The supernatants were stored at -80°C until analysis.

### Statistical methods

All data of hair samples were log-normally distributed (p<0.05); therefore, analyses were performed on log-transformed data. Medians and interquartile ranges (IQR) were used to summarize the results. Pearson correlations were used to assess relationships between parameters. Multivariate linear regression analysis was used to estimate the determinants on the allergen content in horse hair. For this purpose, all breeds with only 1–4 horses per breed were pooled into one group, i.e. ‘other breeds’. Gender was combined with castration status of the animals resulting in three groups: mares, geldings and stallions. The regression models were firstly performed with breed, gender, age, main type of housing, and horse farm as fixed effects. Of these factors, only breed and gender were identified as important and significant determinants for concentrations of HD, Equ c 1 and Equ c 4. Therefore, the data presented herein are the results of regression analyses performed only with breed and gender as covariates. To assess the variability within the allergen concentrations in the hair of individual horses, the results of the subsample of 57 horses were analyzed using Pearson correlation of log-transformed values. Differences in airborne allergen concentrations between Curly Horses and Quarter Horses were tested using the Mann-Whitney U test. Statistical analyses were carried out using GraphPad Prism version 7.03 (column statistics and correlation analyses) and SAS version 9.4 (linear regression analysis). Values of p<0.05 were considered significant.

## Results

### Sensitivity and specificity of sandwich ELISAs

To develop immunoassays for the detection of horse dander antigens and the major allergen Equ c 1, the concentrations of affinity-purified capture antibody, biotinylated detection antibody, and enzyme conjugate were titrated against each other. The combination with the lowest background values and highest sensitivity was chosen as the end condition for each assay. The lower limits of detection based on the protein concentration of the standards were 0.15 ng/mL for horse dander ELISA and 0.1 ng/mL for Equ c 1 ELISA. The modification of the commercial Equ c 4 ELISA resulted in a lower detection limit of 0.02 U/mL.

**Fig 2:**
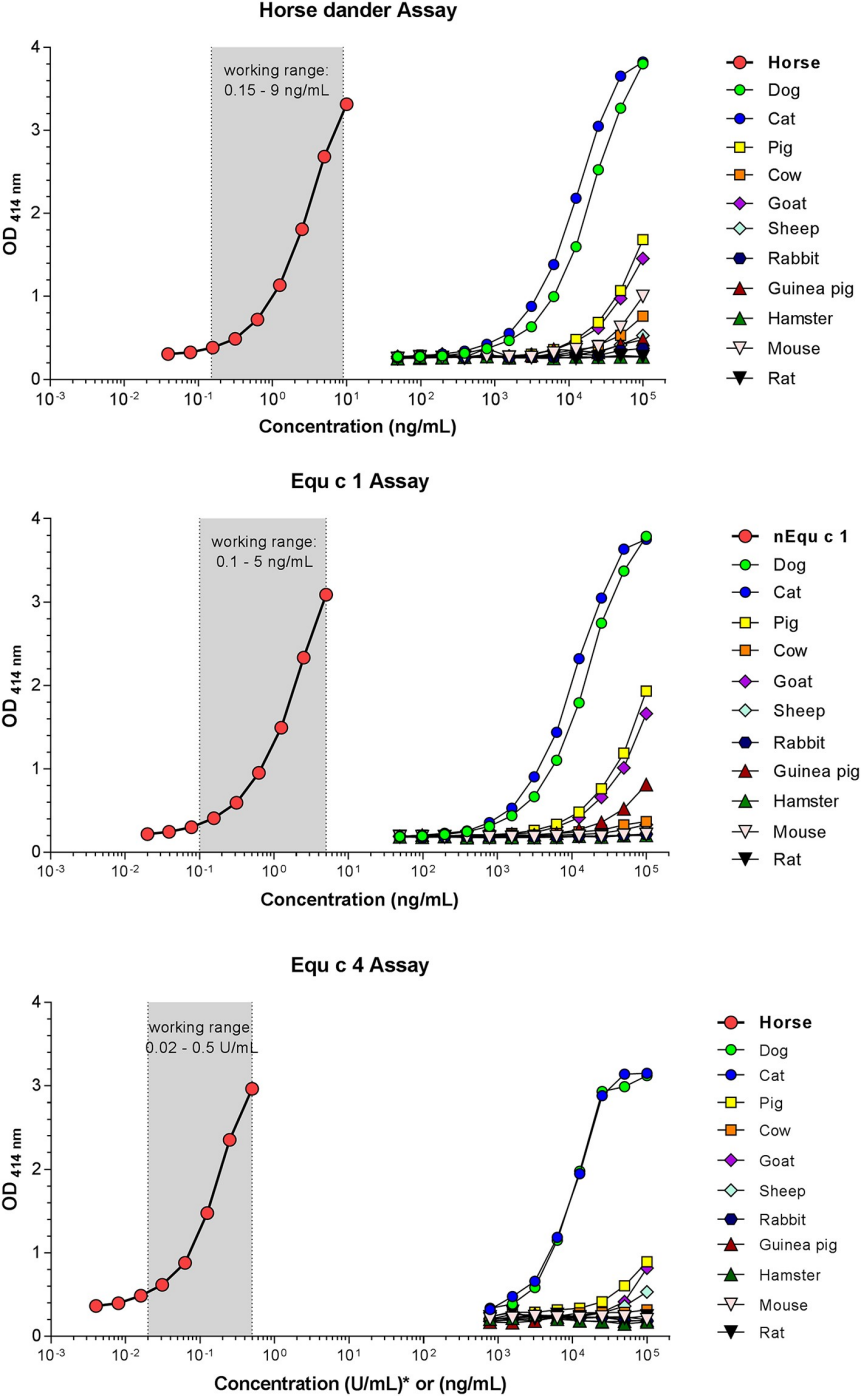
Reactivity of mammalian dander extracts in the horse dander, Equ c 1 and Equ c 4 ELISAs. *U/mL for the horse hair extract.

To investigate the cross-reactivity to other mammalian epithelia antigens, extracts from 11 other species were tested at protein concentrations up to 100 μg/mL in each assay (Fig 2). All three assays showed the strongest reactivity with cat and dog dander extracts. The reactivity curves of both extracts were parallel to the standard curve of the respective assay, with the same intensities obtained at much higher concentrations than with the horse proteins. Cat and dog dander elicited approximately 4000-fold and 6000-fold less reactivity in the horse dander ELISA, and 6000-fold and 8000-fold less reactivity in the Equ c 1 ELISA. In the Equ c 4 assay, cat and dog dander at a concentration of 8000 ng/mL reacted with the same intensity as 0.1 U/mL of the horse standard. Other mammalian epithelia extracts showed only negligible reactions (e.g. pig and goat epithelia) or no reactivity (e.g. rat and rabbit epithelia). No reactions were found with substances that could contaminate horse hair samples, such as molds, mites and grains (tested only in horse dander and Equ c 1 ELISA; S1 Fig).

### Antigen and allergen levels in horse hair

**Fig 3:**
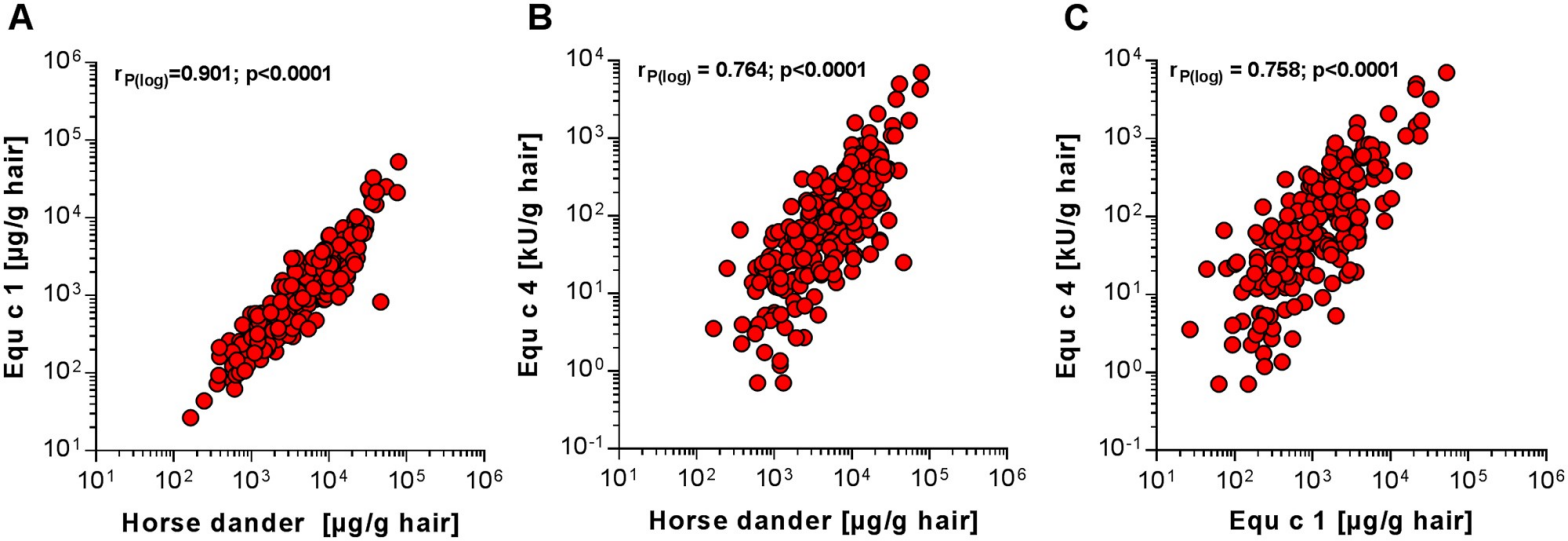
Correlation between results of different quantification assays. A) HD antigen vs. Equ c 1, B) HD antigen vs. Equ c 4, C) Equ c 1 vs. Equ c 4. r_P(log)_: Pearson correlation coefficient of log-transformed values.

In total, 224 hair samples from 32 different horse breeds were investigated with respect to the antigen and allergen content. Log-transformed concentrations were normally distributed. Overall, an enormous variability in antigen and allergen levels was observed between individuals of all breeds. The HD antigen content differed by about 500-fold (165–79335 μg/g, median 4971 μg/g), Equ c 1 content differed by about 2000-fold (27–52936 μg/g, median 1172 μg/g), and the Equ c 4 content differed by about 10000-fold (0.7–7026 kU/g, median 66 kU/g). Significant, positive correlations were observed among HD antigens, Equ c 1 and Equ c 4 levels (Fig 3), with the highest correlation between HD antigen and Equ c 1 values (r_P(log) =_ 0.90, p<0.0001). Equ c 4 correlated weaker with HD antigen, as well as with Equ c 1 (r_P(log) =_ 0.76, p<0.0001 for both).

**Fig 4:**
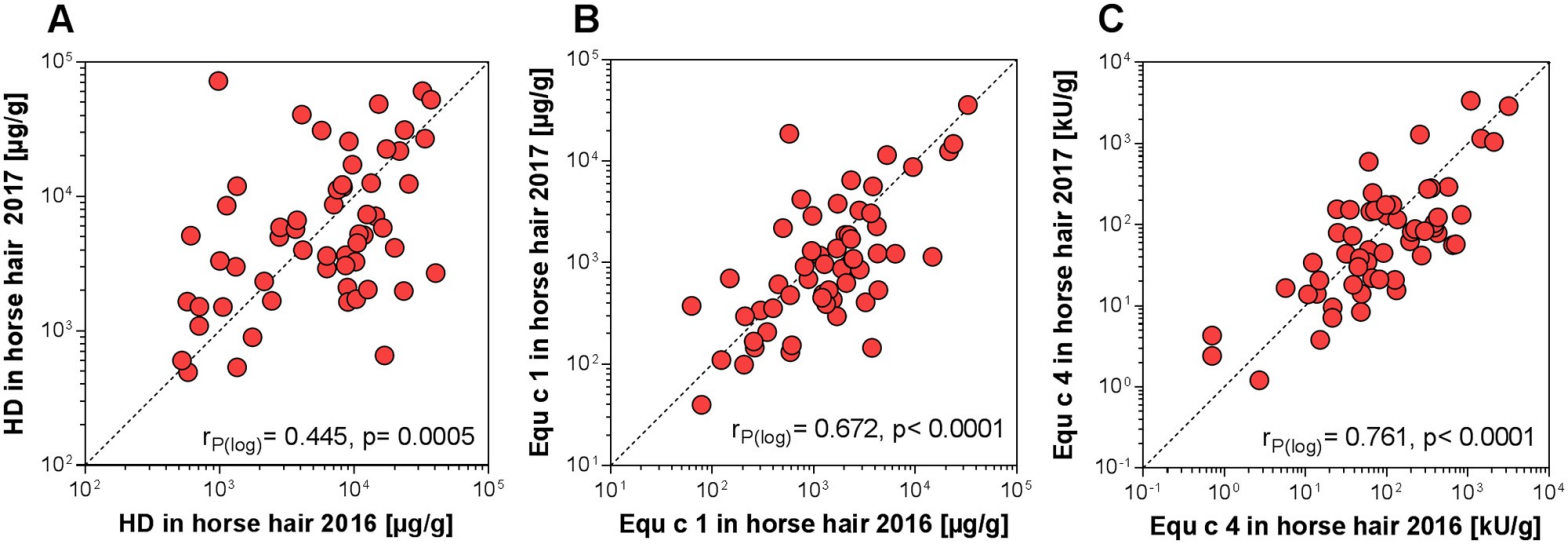
Correlation of A) HD antigen, B) Equ c 1, and C) Equ c 4 concentrations in hair samples collected from the same horses at intervals of one year. r_P(log)_: Pearson correlation coefficient of log-transformed values.

To demonstrate the variability within the antigen/allergen content in the hair of individual horses, hair samples were collected one year later during the same season (change of coat) in a subsample of 57 animals from the original collective. Variations also occurred with the same horses, for example deviations of up to 26-fold were observed for both HD antigen and Equ c 1, and up to 12-fold for Equ c 4. The average of the quotients (sampling 2016/sampling 2017) was 2.3, 2.6 and 2.4 for HD antigen, Equ c 1 and Equ c 4, respectively. Both single allergen levels correlated well and significantly between two samplings (r_P(log) =_ 0.67, p<0.0001 for Equ c 1 and r_P(log) =_ 0.76, p<0.0001 for Equ c 4); whereas, HD antigen values showed poor correlation (r_P(log) =_ 0.44, p = 0.0005) (Fig 4).

The descriptive statistics of antigen and allergen concentrations in horse hair samples are presented in Table 2. “Hypoallergenic” Curly Horses had the second highest concentrations of HD antigens and Equ c 4, and third highest concentrations of Equ c 1 of all breeds. Table 3 summarizes the mutually adjusted results investigating the impact of breed and gender or castration status on antigen and allergen levels. Compared to Curly Horses, most of the investigated breeds had significantly lower levels of all tested parameters. For example, HD antigen, Equ c 1 and Equ c 4 concentrations were about 3-times lower in Quarter Horses. The lowest antigen and allergen levels were detected in the hair of Tinker Horses and Icelandic Horses with similar median values for all three parameters. Both breeds were associated with approximately 7-fold reduced HD antigen and Equ c 1 levels and 20-fold reduced Equ c 4 levels compared to Curly Horses. Oldenburg Horses were the breed with the highest antigen and allergen concentration, but the differences in comparison to Curly Horses were not statistically significant. When classified according to gender, no substantial differences were found between mares and geldings. Compared to mares, significantly higher levels of all three parameters were measured in the hair of stallions. The concentrations of HD antigen, Equ c 1 and Equ c 4 were increased by factors of 2.2, 3.5 and 6.7, respectively. The models explained a large proportion of the total variability in antigen and allergen levels (R^2^_adj_ = 42–46%).

**Table 2:** Concentrations of horse dander antigen, Equ c 1 and Equ c 4 in hair samples according to breed and gender.

	N	Horse dander (μg/g hair)		Equ c 1 (μg/g hair)		Equ c 4 (kU/g hair)	
		Median	IQR	Median	IQR	Median	IQR
**Breed**							
Curly Horse	33	11789	8653–20109	2399	1393–4305	258	122–615
Shetland Pony	31	1553	827–2849	300	181–583	27	17–71
Quater Horse	23	3839	2295–7529	1140	502–2206	100	38–165
German Riding Pony	22	5389	2262–11650	1085	376–2607	45	24–431
Welsh Pony	18	4197	2501–11343	2119	762–3178	83	39–281
Haflinger	13	5880	3259–7819	1809	762–2726	31	16–61
Icelandic Horse	11	1320	633–2439	321	150–449	11	4–16
Oldenburg Horse	10	20078	10283–46010	6003	1907–17194	538	190–1899
Westphalian Horse	9	8730	4212–25703	2813	1027–7426	170	86–370
Tinker Horse	8	1207	650–4399	390	130–526	9	2–22
Hanoverian Horse	8	4816	3104–19245	1641	609–6736	105	44–598
Other breeds	38	4868	2102–10161	1142	509–2259	55	26–149
**Sex**							
Mares	106	5072	1654–10285	1137	416–2391	58	24–162
Geldings	92	3744	1811–10082	917	384–2298	55	20–120
Stallions	26	12073	4998–34239	4216	1735–21284	709	286–1617
**Total**	224	4971	1954–10969	1172	446–2678	66	25–232

N = number of samples; IQR = interquartile range

**Table 3:** Results of the regression models describing the effect of breed and gender on the antigen and allergen concentrations in horse hair.

Effect	Characterisation	Horse dander [μg/g hair]			Equ c 1 [μg/g hair]			Equ c 4 [kU/g hair]		
		Exp(β)	95% CI of Exp(β)	p value	Exp(β)	95% CI of Exp(β)	p value	Exp(β)	95% CI of Exp(β)	p value
**Intercept**		10,618	7605–14825		2218	1553–3168		181	116–284	
**Breed**	Curly Horse	1.00			1.00			1.00		
	Shetland Pony	0.14	0.09–0.22	**<.001**	0.13	0.08–0.21	**<.001**	0.16	0.09–0.30	**<.001**
	Quarter Horse	0.37	0.23–0.61	**<.001**	0.39	0.23–0.67	**0.001**	0.32	0.16–0.63	**0.001**
	German Riding Pony	0.46	0.28–0.78	**0.004**	0.43	0.24–0.74	**0.003**	0.35	0.17–0.71	**0.004**
	Welsh-Pony	0.34	0.20–0.59	**<.001**	0.43	0.24–0.77	**0.005**	0.30	0.14–0.63	**0.002**
	Haflinger	0.54	0.29–1.00	0.051	0.69	0.35–1.34	0.267	0.16	0.07–0.36	**<.001**
	Icelandic Horse	0.14	0.07–0.26	**<.001**	0.15	0.07–0.29	**<.001**	0.05	0.02–0.12	**<.001**
	Oldenburg Horse	1.55	0.79–3.07	0.202	1.71	0.83–3.52	0.147	1.49	0.60–3.73	0.388
	Westphalian Horse	1.05	0.52–2.14	0.885	1.37	0.64–2.92	0.417	1.02	0.40–2.65	0.971
	Tinker	0.13	0.06–0.28	**<.001**	0.16	0.07–0.34	**<.001**	0.04	0.02–0.12	**<.001**
	Hanoverian Horse	0.59	0.28–1.24	0.163	0.78	0.35–1.73	0.537	0.66	0.24–1.79	0.409
	Other breeds	0.49	0.31–0.77	**0.002**	0.50	0.31–0.82	**0.006**	0.31	0.17–0.58	**<.001**
**Sex**	Mare	1.00			1.00			1.00		
	Gelding	0.88	0.66–1.17	0.390	0.92	0.68–1.24	0.577	0.92	0.62–1.34	0.649
	Stallion	2.18	1.42–3.35	**<.001**	3.53	2.24–5.59	**<.001**	6.70	3.76–12.00	**<.001**
**Adjusted R**^**2**^		0.42			0.46			0.46		

β: regression coefficient; CI: confidence interval; R^2^: coefficient of determination. P values <0.05 are printed in bold

### Antigen and allergen levels in airborne samples

**Fig 5:**
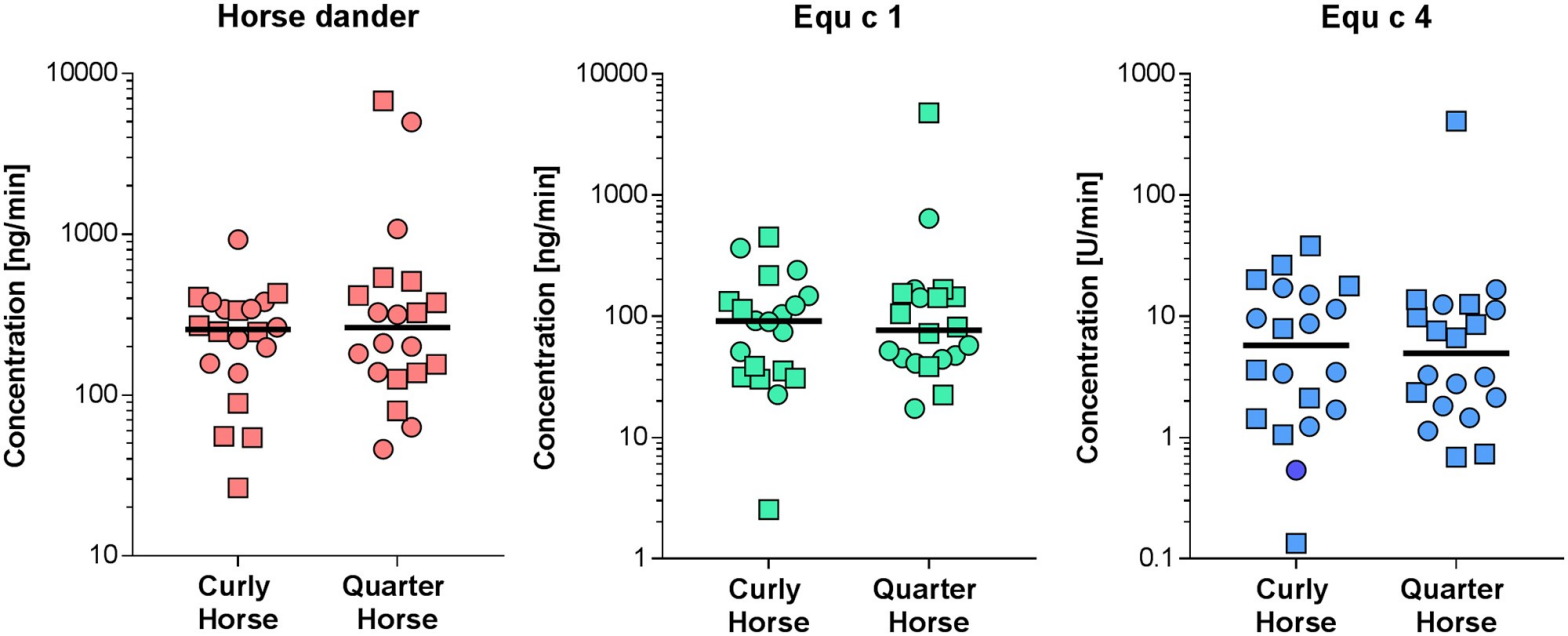
Antigen and allergen concentrations of the nasal filter samples collected during grooming Curly Horses and Quarter Horses. Samples collected by different subjects are marked by squares and circles. Horizontal bars show median values.

HD antigen, Equ c 1 and Equ c 4 concentrations in the personal nasal filter samples collected during grooming horses are presented in Fig 5. No statistically significant differences were found between Curly Horses and Quarter Horses for all tested parameters. For Curly Horses, the median concentration values were: 256 ng/min for HD, 91 ng/min for Equ c 1 and 5.8 U/min for Equ c 4. For Quarter Horses, median concentration values were: 263 ng/min for HD, 77 ng/min for Equ c 1 and 5.0 U/min for Equ c 4. Sorted by subjects that collected the samples, there were also no statistically significant differences between and within the breeds for all determinants. Compared to samples collected during the grooming of horses, the ambient allergen concentrations (without grooming) at different locations of the horse farm were on average 50-fold lower (p <0.0001), with median values of 5.2 ng/min for HD, 1.2 for Equ c 1 and 0.1 U/min for Equ c 4.

## Discussion

The aim of the present study was to compare the antigen/allergen content in horse hair among different breeds with a main focus on the hypoallergenic Curly Horses. So far, no studies have been conducted on the quantitative analysis of allergens in horse hair.

Currently, almost all measurements of mammalian dander allergens are based on the detection of major allergens of each species, e.g. Fel d 1 for cat, Can f 1 for dog, Bos d 2 for cow or Mus m 1 for mouse. For horse allergens, there is a commercial assay that detects the latherin Equ c 4. Although one study has reported that a major fraction of horse sensitized subjects reacted to Equ c 4 [20], this protein is still considered a minor allergen and is not a specific marker of horse sensitization [26]. Therefore, we developed two new immunoassays that are based on polyclonal antibodies against: 1) all proteins present in horse dander extract, and 2) the major horse allergen Equ c 1. In this project, the assays were used for quantitative allergen analysis of horse hair. In the future, the assays can be used for exposure assessment studies to examine the spread of horse allergens in different environments. In the specificity testing, both assays showed some cross-reactions with cat and dog dander extracts. It is very likely that this cross-reactivity is derived from the homologue cat and dog lipocalins Fel d 4 and Can f 1. Equ c 1, Fel d 4 and Can f 1 display high sequence similarities (73–80%) and identities (55–67%), and may contribute to polysensitization and symptoms in individuals allergic to mammals [27,28]. However, the reactivity to cat and dog dander in both assays was very weak (0.01–0.03% reactivity of the protein content). The commercial assay based on monoclonal antibodies against Equ c 4 also showed some reactivity with cat and dog dander. The possible cause of this cross-reaction can be the recognition of latherin-like proteins in mammalian dander extracts. For cat, a latherin-like minor allergen, Fel d 8 has been already characterized [29], but no homologues protein has thus far been identified for dog.

The analysis of 224 hair samples from 32 different horse breeds revealed an enormous variability in antigen and allergen content among individual animals (e.g up to 10,000-fold for Equ c 4). Similar results had been previously obtained in investigations of dog or bovine hair samples. The content of the major dog allergen, Can f 1 differed up to four orders of magnitude [30,31], and the major bovine allergen, Bos d 2 varied up to three orders of magnitude among individual animals [24]. A wide variation in allergen concentrations was also observed among animals of the same breed from each species. These findings imply that allergen production is strongly influenced by individual factors, and some animals might be classified as either high or low allergen producers. The repeated collection and investigation of hair samples from the same horses in our study confirm this hypothesis. The results correlated well and significantly between the two samplings, especially for the single allergens Equ c 1 and Equ c 4. Although allergen content in hair collected from a single horse varied, the differences were small (on average 2.5-fold) relative to the variability within the breed or among all individuals. Therefore, we conclude that the allergen production remains reasonably constant within an individual. Similar results have been also reported for cats. Wentz et al. investigated the variability of allergen shedding by measuring of Fel d 1 in air samples collected in a lucite chamber over the course of several days [32]. In general, the high producers’ samples remained high in allergen content, and the low producers’ samples remained low.

Despite the high variability among individuals within and among the different breeds, this study showed for the first time that antigen and allergen levels in horse hair are significantly related to breed. Paradoxically, the so-called hypoallergenic Curly Horses had significantly higher HD antigen, Equ c 1 and Equ c 4 levels in hair than the majority of other investigated breeds. These results are in accordance with previous studies that have shown significant differences in Can f 1 levels among dog breeds [30,31]. Both studies have found that Poodles had the highest and Labrador Retrievers had the lowest Can f 1 concentrations in hair. Moreover, a study by Vredegoor et al. showed significantly higher Can f 1 levels in hair of hypoallergenic dogs, such as Labradoodle, Poodle, Spanish Waterdog, and Airedale terrier (geometric mean (GM), 2.26 μg/g) compared to hair from non-hypoallergenic dogs (GM, 0.77 μg/g) [31]. In contrast, there were no significant differences in Bos d 2 levels in bovine hair among different breeds [24].

In our study, the horse antigen and allergen content in hair was also significantly related to the gender combined with the castration status of male animals. Whereas mares and geldings did not differ significantly, stallions displayed significantly higher concentrations of all measured parameters. With respect to sex and castration differences, contradictory results have been published for different mammalian species so far. In the study by Vredegoor et al., gender (male vs. female) had no significant effect on Can f 1 levels, but castration status (sex of the animals not specified) was associated with reduced Can f 1 levels in hair [31]. In contrast, Ramadour et al. observed significantly higher Can f 1 concentrations in the hair of male dogs compared to female dogs, although the difference was small (11.75 vs. 8.89 μg/g, p = 0.037) [30]. On the other hand, no statistically significant difference was found between castrated and non-castrated dogs for both females and males [30]. In bovine hair samples, Bos d 2 levels did not differ between male and female animals, but the castration status was not noted in this study [24]. In another study, Fel d 1 levels were significantly higher in fur washes collected from male cats compared to female cats (151 vs. 751 mU/ml, p = 0.023) [33]. Castration of male cats significantly decreased production of Fel d 1, and injections of testosterone could reverse this phenomenon [34]. Male rodents have been shown to excrete up to 100-fold higher levels of urinary allergens (Mus m 1 or Rat n 1) than female rodents [35,36]. In summary, these results suggest that the production of major allergens appear to be higher in male, non-castrated animals, which is in agreement with our findings for stallions. However, stallions are mostly used for breeding or for competitive sports and much less for leisure activities. Thus, the higher allergen content in stallions is of poor relevance for the majority of exposed people.

Another possible reason for the better tolerance of horse allergic subjects to Curly Horses by could be a reduced release of allergens into the air. Because of the increased sebum content on fur, the allergens could better adhere to the hair, thus less become airborne. However, our measurements of airborne concentrations of HD antigen, Equ c 1 and Equ c 4 during grooming horses failed to demonstrate a lower release of allergens during grooming of Curly Horses. All three tested parameters did not differ between Curly Horses and Quarter Horses, which are not considered to be hypoallergenic. Taking into account that Curly Horses have on average 3 times higher allergen content in hair compared to Quarter Horses, one might assume that the percentage of released allergens into the air is lower in Curly Horses. Nevertheless, the level of inhaled allergens during grooming of Curly or Quarter Horses remains approximately the same. Furthermore, in our study no long-term exposure assessment and no measurements during riding or other activities have been performed.

There was also no evidence for reduced shedding of allergens into the environment by so-called hypoallergenic dogs. In settled floor dust, as well as in settled airborne dust collected using EDC (electrostatic dust fall collector), no differences in Can f 1 concentrations were found between houses with hypoallergenic and non-hypoallergenic dogs [31,37]. These results refer to studies that represent long-term exposure assessment in contrast to our study with nasal filters.

In the present study, personal nasal filters were used as a novel collection tool for airborne dust. Already after wearing the filters for 10 min, horse allergens were detectable in all samples. Due to its low cost and ease of use, this method is a desirable alternative compared to the active collection of airborne dust on filters using pumps. Conventional airborne dust sampling requires expensive equipment and trained staff. The pumps are noisy and need recharging and calibration. The usage of pumps, particularly for the airborne measurement of animal allergens, may be disadvantageous because their noise levels may irritate animals. However, the nasal filter sampling method has several limitations. Allergen levels can only be presented in ng/min and not in ng/m³, and the size of sampled particles is poorly studied. This makes comparison with other studies that collect allergens using personal air samplers with constant flow rates and with defined dust fractions difficult. Moreover, sampling time is limited and the necessity of nasal breathing complicates application during heavy work [38].

The supposed hypoallergenic properties of Curly Horses could neither be explained by decreased allergen levels in hair nor by a reduced allergen release into the air during grooming. However, we cannot exclude that a hypoallergenic effect really exist for this special breed. Previous studies designed to analyze reduced symptoms in allergic riders concluded that purebred Curly Horses may be a suitable alternative for horse allergic people who want to continue riding [21,22]. Although mild allergic reactions have been initially observed in some riders, these reactions decreased the longer the subjects were in contact with Curly Horses. Additionally, a loss of reactivity to normal horses was also observed in some riders indicating an induction of immune tolerance. However, it should be noted that these studies lack the controls with other horse breeds. It is possible that the same effect could be achieved by gradually increasing contact to “normal” horses. Nevertheless, there are several websites, as well as newspaper and TV posts reporting stories of horse allergic people who can handle Curly Horses without suffering any allergic reactions. Similar health effects have been seen in subjects with dog or cat allergies as well. The owners often report no allergic reaction to their own pet, but only to other animals. Psychological factors may contribute to these phenomena. Curly Horses, based on their unique habitus, or the own pet may be classified as a “safe” exposure. It is possible that mental attitude could lead to altered perception or even suppression of allergic symptoms.

## Conclusion

This study showed for the first time that antigen and allergen levels in horse hair are significantly related to breed and non-castrated male animals, although immense variability (up to 4 orders of magnitude) among individuals was observed. No evidence was found for a reduced production of allergens by so-called hypoallergenic Curly Horses. In addition, no evidence was found for a reduced release of allergens from Curly Horses’ fur into the air during grooming. In contrast, Curly Horses had significantly higher allergen levels in hair compared to several control breeds and the airborne allergen concentrations did not differ between Curly Horses and non-hypoallergenic Quarter Horses. These results provide no scientific evidence to suggest that Curly Horses have hypoallergenic properties. The reasons why horse allergic patients have fewer or even no symptoms while handling this special breed remain unclear and need further investigation.

## Supporting information

S1 Fig Reactivity of mite (A and B), mold (C and D) and grain extracts (E and F) in the horse dander ELISA (A, C, E) and Equ c 1 ELISA (B, D, F). (TIF)
