# Supplementary figures and images for: Lower allergen levels in hypoallergenic Curly Horses? A comparison among breeds by measurements of horse allergens in hair and air samples
Source: PLoS One. 2018 Dec 12;13(12):e0207871. doi: 10.1371/journal.pone.0207871 (PMC6291085; doi:10.1371/journal.pone.0207871)

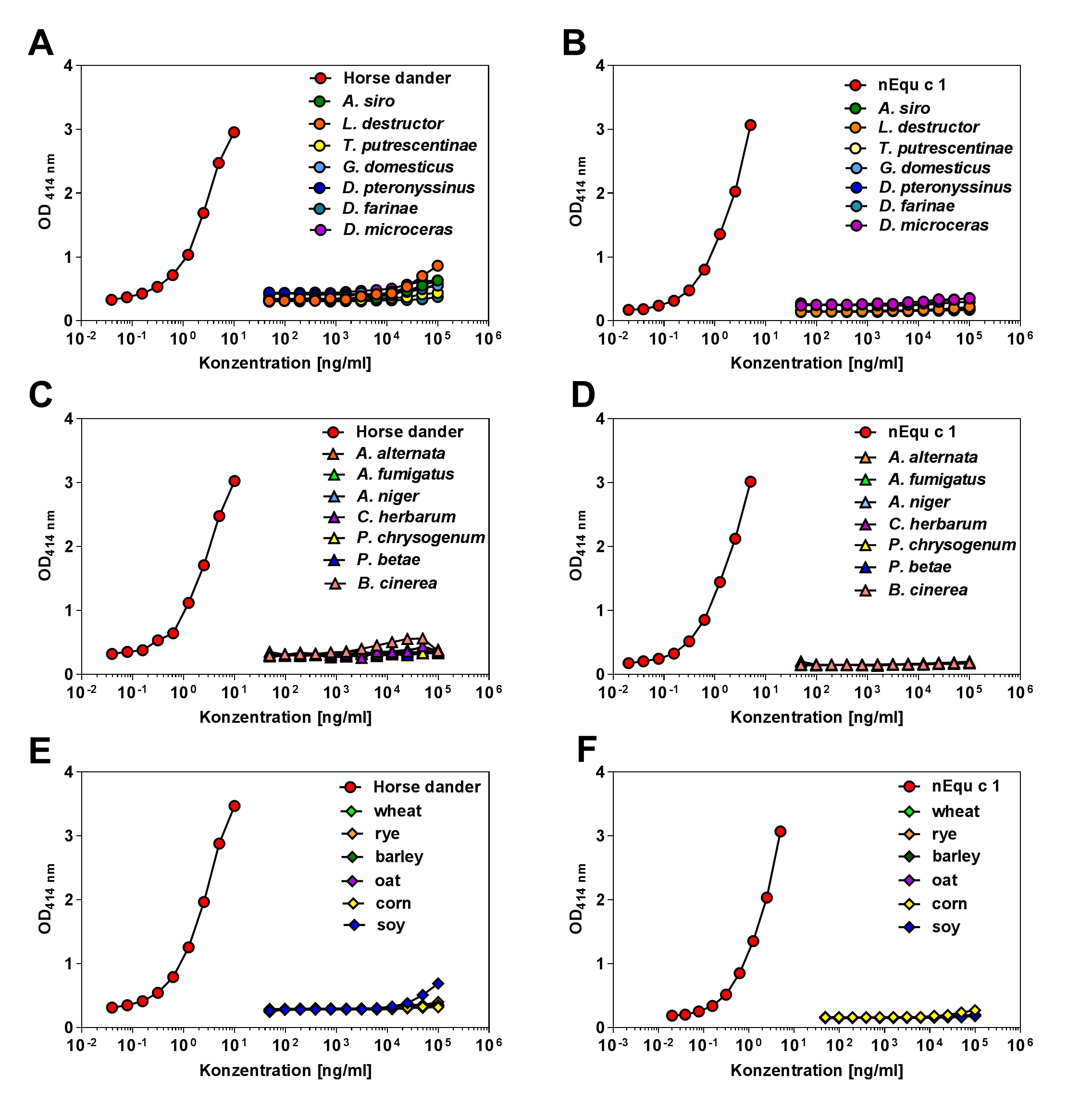

Supplement: S1 Fig — (TIF) [file pone.0207871.s001.tif]
